# Assessment of 3-Cyanobenzoic Acid as a Possible Herbicide Candidate: Effects on Maize Growth and Photosynthesis

**DOI:** 10.3390/plants14010001

**Published:** 2024-12-24

**Authors:** Luiz Henryque Escher Grizza, Isabela de Carvalho Contesoto, Ana Paula da Silva Mendonça, Amanda Castro Comar, Ana Paula Boromelo, Ana Paula Ferro, Rodrigo Polimeni Constantin, Wanderley Dantas dos Santos, Rogério Marchiosi, Osvaldo Ferrarese-Filho

**Affiliations:** Laboratory of Plant Biochemistry, Department of Biochemistry, State University of Maringá, Maringá 87020-900, Brazil; luizheg@gmail.com (L.H.E.G.); isabelacontesoto@gmail.com (I.d.C.C.); silvamendoncaanapaula@gmail.com (A.P.d.S.M.); amandacomar9@gmail.com (A.C.C.); paulaboromelo123@gmail.com (A.P.B.); apferro2@uem.br (A.P.F.); rpconstantin@uem.br (R.P.C.); wdsantos@uem.br (W.D.d.S.)

**Keywords:** chlorophyll *a* fluorescence, enzyme inhibitor, gas exchange, phenolics, phytotoxicity, pyruvate *O*-phosphate dikinase, weeds

## Abstract

Chemical weed control is a significant agricultural concern, and reliance on a limited range of herbicide action modes has increased resistant weed species, many of which use C4 metabolism. As a result, the identification of novel herbicidal agents with low toxicity targeting C4 plants becomes imperative. An assessment was conducted on the impact of 3-cyanobenzoic acid on the growth and photosynthetic processes of maize (*Zea mays*), a representative C4 plant, cultivated hydroponically over 14 days. The results showed a significant reduction in plant growth and notable disruptions in gas exchange and chlorophyll *a* fluorescence due to the application of 3-cyanobenzoic acid, indicating compromised photosynthetic activity. Parameters such as the chlorophyll index, net assimilation (*A*), stomatal conductance (*g*_s_), intercellular CO_2_ concentration (*C*_i_), maximum effective photochemical efficiency (F_v′_/F_m′_), photochemical quenching coefficient (q_P_), quantum yield of photosystem II photochemistry (ϕ_PSII_), and electron transport rate through PSII (ETR) all decreased. The *A*/PAR curve revealed reductions in the maximum net assimilation rate (*A*_max_) and apparent quantum yield (ϕ), alongside an increased light compensation point (LCP). Moreover, 3-cyanobenzoic acid significantly decreased the carboxylation rates of RuBisCo (V_cmax_) and PEPCase (V_pmax_), electron transport rate (*J*), and mesophilic conductance (*g*_m_). Overall, 3-cyanobenzoic acid induced substantial changes in plant growth, carboxylative processes, and photochemical activities. The treated plants also exhibited heightened susceptibility to intense light conditions, indicating a significant and potentially adverse impact on their physiological functions. These findings suggest that 3-cyanobenzoic acid or its analogs could be promising for future research targeting photosynthesis.

## 1. Introduction

C4 plants, such as maize (*Zea mays*), sugarcane (*Saccharum officinarum*), millet (*Pennisetum glaucum*), and sorghum (*Sorghum bicolor*), have evolved highly specialized photosynthetic pathways that enable them the competitive ability to capture and use atmospheric CO_2_. This efficiency is facilitated by the actions mesophyll and bundle sheath cells working in the C4 carbon concentration mechanism [1]. More specifically, the assimilation and reduction of CO_2_ occur in two distinct steps, implemented by different enzymes. Initially, CO_2_ is assimilated in the cytoplasm of mesophyll cells, where it is converted into bicarbonate, then incorporated into phosphoenolpyruvate to form oxaloacetate by the action of phosphoenolpyruvate carboxylase (PEPCase). The oxaloacetate is then converted into malate or aspartate, which are transported to the bundle sheath cells. In these cells, malate or aspartate is decarboxylated, releasing CO_2_, which is in turn captured by RuBisCo for further assimilation [1]. Because the high-CO_2_ environment, the carboxylative activity of RuBisCo increases and reduces oxygen interference, minimizing photorespiration [2,3]. As a result, C4 plants grow faster and accumulate more biomass, especially in areas with high temperature and high light. The ability of C4 plants to concentrate CO_2_ provides them with a significant advantage in challenging climates, making them essential for food, feed, and also bioenergy production [4]. In addition, C4 plants contribute to about 18% of global plant productivity, highlighting their essential role in both ecosystems and agriculture [5].

Agroecosystems, much like any other ecosystem, are greatly influenced by their surrounding environment and climate, which in turn impacts the various living organisms within them. Since the primary goal of an agroecosystem is to meet specific human needs, not all organisms within it are desirable. For instance, pests, diseases, and weeds are typically unwelcome. Weeds are common in all agricultural settings and can significantly reduce both productivity and crop quality. Climate change, with higher CO_2_ levels, rising temperatures, and changing rainfall patterns, directly impacts agriculture, favoring the growth of competitive C4 weeds [6]. Weeds present a significant challenge due to their rapid growth, the ability to absorb resources, and the interception of sunlight, all of which can disrupt the agroecosystem. To address these challenges, integrated weed management strategies—such as crop rotation, selective herbicide use, and other agronomic practices—are essential [7,8].

Herbicides are a primary tool for weed control, but their repeated use often leads to the emergence of resistant weed biotypes. These resilient weeds not only survive but thrive, dominating plant populations and creating new challenges. Atrazine, a pre-emergent herbicide widely used in maize cultivation, exemplifies this challenge. It inhibits photosystem II, disrupting photosynthesis after being absorbed primarily by plant roots [9]. However, its extensive use has raised significant concerns due to its persistence in the environment, leaching into groundwater and surface water, and its long half-life. These characteristics, combined with evidence of adverse health effects, including carcinogenicity and endocrine disruption, led to its ban in the European Union [10]. Despite this, atrazine remains one of the most commonly used herbicides in countries like Brazil [11]. The environmental and health concerns associated with herbicides such as atrazine, coupled with the rise of resistant weed biotypes, emphasize the urgent need for novel herbicides with unique mechanisms of action [12]. These alternatives must balance efficacy and environmental safety to ensure sustainable weed management practices. Currently, there are 533 documented cases of herbicide-resistant weeds worldwide, involving 273 species (156 dicots and 117 monocots). These resistant weeds have evolved to survive against 21 out of 31 known herbicide sites of action and 168 different herbicides, impacting 101 crops in 72 countries. Some species have developed resistance to up to seven herbicide sites of action, highlighting the significant challenge posed by resistant weeds in the *Poaceae* family, which contains 92 resistant species—double that of the second most affected family, *Asteraceae* [9]. Many of the most problematic weed species are C4 plants [13], with 14 of the 18 most noxious species belonging to this group [14]. The continuous rise of herbicide-resistant weeds, coupled with the lack of new herbicides with novel modes of action since 1980 [12], highlights the pressing need for more effective, selective, and environmentally friendly herbicide solutions.

Our research team is dedicated to identifying and evaluating novel herbicides with innovative modes of action. We utilize a combination of in silico, in vitro, and in vivo approaches to assess the effectiveness of enzyme inhibitors with potential herbicidal activity [15,16]. Targeting enzymes essential for C4 photosynthesis, such as pyruvate *O*-phosphate dikinase (PPDK), could be one promising strategy to develop herbicides [17,18]. Inhibiting PPDK, a crucial enzyme in mesophyll cells, has been shown to reduce photosynthetic activity, potentially providing a method for controlling C4 weeds [19,20,21]. Enzymatic inhibitors, which downregulate target enzymes, play a critical role in controlling plant metabolism. Because PPDK is essential for C4 plants to assimilate CO_2_ through photosynthesis, we evaluate selective inhibitors of this enzyme as potential candidates for new herbicides. We have already tested a few compounds, selected from a virtual library and anchored to the PPDK active site of *Digitaria sanguinalis*, to develop specific PPDK inhibitors. Using virtual screening and drug repositioning approaches, we found five promising chemicals (bromoacetic, bromopyruvic, 2-bromobutyric, 2-bromopropionic, and 3-cyanobenzoic acids) as potential PPDK inhibitors. Among these, bromoacetic acid showed a dose-dependent inhibition of PPDK activity in maize, significantly affecting gas exchange and reducing the photosynthetic rate [22]. These findings laid the groundwork for further exploration of PPDK inhibitors. More recently, we found that 3-cyanobenzoic acid not only inhibited maize seedling growth but also triggered oxidative stress, increasing reactive oxygen species (ROS) production [23]. Despite the lack of literature on this specific molecule, 3-cyanobenzoic acid is a simple phenolic compound, part of a class of molecules derived from benzoic and cinnamic acids, which are known for their extensive biological activities [24]. These include allelopathic interactions between plants, where these simple phenols can affect growth by disrupting electrical conductivity in membranes, hormonal balance, and stomatal closure, and have been shown to impact structural components of mitochondria and chloroplasts as primary targets [24]. Notably, this compound can also be synthesized by bacteria, presenting a promising opportunity for its bio-sourced production, which could enhance its sustainability and commercial viability [25]. Furthermore, 3-cyanobenzoic acid is like certain O-group herbicides, such as chloramben, dicamba, naptalam, and dimethyl-2,3,5,6-tetrachloroterephthalate, which are herbicides known for mimicking auxin and inhibiting microtubule assembly [9,26].

Based on our earlier findings, we hypothesized that the adverse impacts of 3-cyanobenzoic acid extend beyond root damage and that this compound could potentially serve as a targeted herbicide for C4 plants. Thus, this study aimed to evaluate the phytotoxic effects of 3-cyanobenzoic acid on maize (*Zea mays*), focusing on its impact on growth, photosynthetic processes, and biochemical parameters. Maize was selected as a model plant due to its C4 metabolism and ease of cultivation. To evaluate enzyme activity and photochemical efficiency, we measured intercellular CO_2_ concentration (*C*_i_) and constructed irradiance light curves. Chlorophyll *a* fluorescence emission, a noninvasive and quantitative method, was used to monitor changes in photosynthetic processes. From the fluorescence data, we calculated key parameters such as photochemical yield, electron transport ability, and energy dissipation. We also generated OJIP curves and conducted the JIP fluorescence test to analyze energy flow through reaction centers and photosystems, providing a comprehensive assessment of the compound’s effects on plant photosynthetic performance. By assessing these effects, we aim to explore the potential of 3-cyanobenzoic acid as a candidate herbicide targeting C4 plants.

## 2. Materials and Methods

The maize (*Zea mays* L. cv. IPR-164) seeds were cleaned by soaking in a 2% sodium hypochlorite for three minutes, followed by thorough rinsing with deionized water to ensure cleanliness. The seeds were then evenly spread on pre-moistened germination paper (Germitest^®^ CEL-060, Netlab, São Paulo, Brazil) and covered with another sheet, rolled up, and kept in the dark at 25 °C for three days to promote germination. Germinated seedlings with radicle lengths between 5 and 6 cm were randomly selected and grouped into sets of 12, then transferred to hydroponic systems. These seedlings were placed in Styrofoam trays and placed in glass containers filled with 200 mL of ^1^/_4_ strength nutrient solution at pH 6.0 [27], which contained either 0 (control), 0.5, or 1.0 mM of 3-cyanobenzoic acid (Sigma–Aldrich, St. Louis, MO, USA). The seedlings were grown under controlled conditions with a 12 h light/12 h dark photoperiod at 25 °C and an irradiance of 300 μmol photons m^−2^ s^−1^. The nutrient solution was replaced every other day to maintain optimal growth conditions. On the 14th day (V2 stage) entire plants were carefully harvested for biometric measurements, while the second fully expanded leaf was sampled for further biochemical and physiological analyses.

### 2.1. Biometric Measurements

Plant height was determined by measuring the distance from the point of seed attachment to the culm up to the base of the last fully developed leaf and the main root. Stem diameter was precisely measured at the first internode using a digital caliper. Fresh biomass of shoots and roots was assessed through gravimetric analysis. Leaf area estimation was performed using the Easy Leaf Area Free 1.02 software for mobile devices [28]. These measurements ensured consistency and accuracy, with a total of 18 plants evaluated per treatment.

### 2.2. SPAD Index, Chlorophyll a Fluorescence, and Gas Exchange

Chlorophyll content was measured using a SPAD-502 m (Konica Minolta^®^, Ramsey, NJ, USA). Chlorophyll *a* fluorescence parameters were assessed with a pulse-amplitude modulated fluorometer (PAM) integrated into an LI-6800 portable photosynthesis system (Li-Cor^®^, Lincoln, NE, USA). Measurements were conducted in the middle region of the last fully expanded leaf, previously dark-adapted for 10 h to fully oxidize photosystem II (PSII), ensuring optimal data accuracy [29]. Key fluorescence parameters were recorded, including minimal fluorescence intensity (F_0_), observed in the absence of light when PSII reaction centers were fully open, and maximal fluorescence intensity (F_m_), elicited under a high-intensity multiphase flash of 8000 µmol m^−2^ s^−1^ when PSII centers were fully closed. After that, the maximum photochemical efficiency (F_m_ − F_0_/F_m_) of the plants was calculated [30]. A total of eighteen plants were evaluated for each treatment.

Next, measurements focused on light-adapted leaf regions under controlled conditions (irradiance of 1400 μmol m^−2^ s^−1^ and CO_2_ concentration of 400 µmol mol^−1^) to achieve complete photosynthetic induction. Using the PAM fluorometer and the LI-6800 infrared gas analyzer (IRGA, Li-Cor^®^), fluorescence and gas exchange parameters were recorded. Variables such as net assimilation (*A*), stomatal conductance (*g*_s_), maximum quantum yield of PSII photochemistry (F_v′_/F_m′_), effective quantum yield of PSII photochemistry (ϕ_PSII_), electron transport rate (ETR), photochemical quenching coefficient (q_P_), and non-photochemical quenching (NPQ) were recorded. The measurements were performed at 27 °C; relative humidity of 60%; air flow of 700 µmol^−1^; CO_2_ concentration of 400 µmol^−1^; photosynthetically active radiation (PAR) of 1400 µmol m^−2^ s^−1^; and multiphase saturating light flash of 8000 µmol m^−2^ s^−1^. Values were recorded from duplicated points obtained when constructing light curves and *A*/*C*_i_ curves, which are explained in detail in the following sections. The values were recorded point by point with adaptation times ranging from 60 to 120 s after changing the parameters associated with the curve (light or CO_2_), indicating the steady state of the leaf area when the instantaneous rate of change of water, CO_2_ concentration, and chlorophyll *a* fluorescence was varied in the recorded values less than one in the device sample chamber. A total of 22 plants were evaluated per treatment, ensuring high reproducibility and reliability of the results.

### 2.3. Net Assimilation Curves in Response to Changes in Photosynthetically Active Radiation (A/PAR)

Plants were light-adapted under controlled conditions (1400 μmol m^−2^ s^−1^ irradiance and a fixed CO_2_ concentration of 400 µmol mol^−1^ within the sample chamber) until full photosynthetic induction was achieved. Other environmental parameters were maintained as previously described to ensure consistency. The net assimilation rate (*A*) curve was generated by systematically recording photosynthetically active radiation (PAR) values at a series of pre-defined intervals using the LI-6800 IRGA. Measurements at each PAR level were taken over 60 to 120 s, allowing the plants to reach a stable photosynthetic state before data collection. PAR levels ranged from 2000 to 0 μmol photons m^−2^ s^−1^, following a descending sequence of 2000, 1800, 1600, 1400, 1200, 1000, 800, 600, 400, 200, 175, 150, 125, 100, 75, 50, 25, and 0 μmol photons m^−2^ s^−1^. This comprehensive range ensured an accurate representation of the light response curve. Measurements were taken from four plants per treatment.

The resulting *A*/PAR curve was well-suited to the light range of 0 to 2000 μmol m^−2^ s^−1^, with particular emphasis on the linear phase observed between 0 and 175 μmol m^−2^ s^−1^. Key parameters derived from the curve included the maximum net assimilation rate (*A*_max_), apparent quantum yield (ϕ), light compensation point (LCP), and dark respiration rate (*R*_D_). These parameters were calculated using a Microsoft^®^ Excel-based tool developed by Lobo et al. [31], ensuring precision in data analysis and parameter estimation.

### 2.4. Net Assimilation Curves in Response to Changes in Intercellular CO_2_ Concentration (A/C_i_)

Like the *A*/PAR curve measurements, acclimatization of the plants to light intensity at 1400 µmol m^−2^ s^−1^ was ensured prior to data collection. The recording time was standardized to capture the steady-state photosynthetic responses of the plants. During the experiment, the light intensity was maintained at a constant PAR of 1400 µmol m^−2^ s^−1^, while the concentration of available CO_2_ was systematically varied. The measurements began at a CO_2_ concentration of 400 µmol mol^−1^, progressively decreasing in steps without reaching zero. Following this, CO_2_ levels were increased in the sequence: 400, 200, 25, 50, 75, 100, 125, 150, 175, 200, 225, 250, 270, 300, 325, 350, 400, 400, 500, 600, 700, 800, 1000, 1200, 1400, 1600, 1800, and 2000 µmol mol^−1^. This method ensured comprehensive profiling of photosynthetic responses at varying CO_2_ concentrations. Measurements were performed on six plants per treatment, providing robust and replicable data [2,32]. To analyze the results, spreadsheets developed by Zhou et al. [2] were used to fit assimilation curves based on the concentration of intercellular CO_2_, tailored specifically for C4 plants. From these fitted curves, key photosynthetic parameters were derived, including the maximum RuBisCo carboxylation rate (V_cmax_), maximum PEPCase carboxylation rate (V_pmax_), photosynthetic electron transport rate (*J*), and mesophilic conductance (*g*_m_).

### 2.5. Transient Responses of the Chlorophyll a Fluorescence (OJIP Curve) and Derived Parameters (JIP Test) Parte Superior do Formulário

Fast fluorescence kinetics were measured using the inductive flash feature of the LI-6800 IRGA PAM fluorometer (Li-Cor^®^, Lincoln, NE, USA) in plants that had been dark-adapted for 10 h to ensure fully open PSII reaction centers. Fluorescence emissions were recorded at millisecond intervals for one second, capturing the rapid changes associated with the OJIP transient. Data from 18 plants per treatment were collected to produce OJIP curves, with time converted to a logarithmic scale, showcasing different stages between O (F_0_) and P (F_M_) phases. The photochemical phase (O–J) reflects the reduction of the primary quinone acceptor (Q_A_), while the thermal phase (J–I) corresponds to electron transfer between Q_A_ and the secondary quinone acceptor (Q_B_). The I–P phase represents fluorescence emissions driven by the pool of oxidized plastoquinone molecules [33,34,35]. This detailed division of phases enables a comprehensive understanding of electron transport dynamics within the photosynthetic apparatus.

Fluorescence data were analyzed using the JIP test, which translates the OJIP curves into specific energy fluxes and parameters indicative of photosynthetic efficiency. Key energy fluxes included absorption per reaction center (ABS/RC), trapped energy per reaction center (TR_0_/RC), electron transport flux per reaction center (ET_0_/RC), dissipated energy per reaction center (DI_0_/RC), and the density of active PSII reaction centers per cross-section (RC/CS). These metrics provided insights into energy dynamics and the operational efficiency of PSII. The maximum quantum yield of primary photochemistry (TR_0_/ABS), quantum yield for electron transport from Q_A_ to Q_B_ (ET_0_/ABS), and the probability that a trapped exciton moves an electron into the electron transport chain beyond Q_A_^−^ (ET_0_/TR_0_) were also assessed. Two critical performance indices, PI_ABS_ and PI_TOTAL_, were derived. PI_ABS_ quantifies the conservation of absorbed energy from PSII up to Q_B_ reduction, while PI_TOTAL_ is an indicator of the conservation of energy absorbed by the PSII until the decline in the final PSI acceptors) [33,34,35]. Together, these indices provide an integrated evaluation of the energy conservation ability within the photosynthetic electron transport chain, offering a robust framework for assessing plant photosynthetic performance under varying treatments.

### 2.6. Statistical Analysis

The experimental design used was completely randomized, and the data were submitted to one-way analysis of variance (ANOVA). Significant differences were then compared using Dunnett’s test at both 5% and 1% levels of significance. Data from the *A*/PAR and *A*/*C*_i_ curves were adjusted using Microsoft^®^ Excel spreadsheet provided by Lobo et al. [31] and Zhou et al. [2]. Furthermore, parameters derived from the JIP test were calculated using data from the chlorophyll *a* transient curve, normalized, and subjected to the same tests. All statistical analyses were performed using GraphPad Prism^®^ program, version 8 (La Jolla, CA, USA).

## 3. Results

### 3.1. Effects on Biometric Measurements

**Figure 1:**
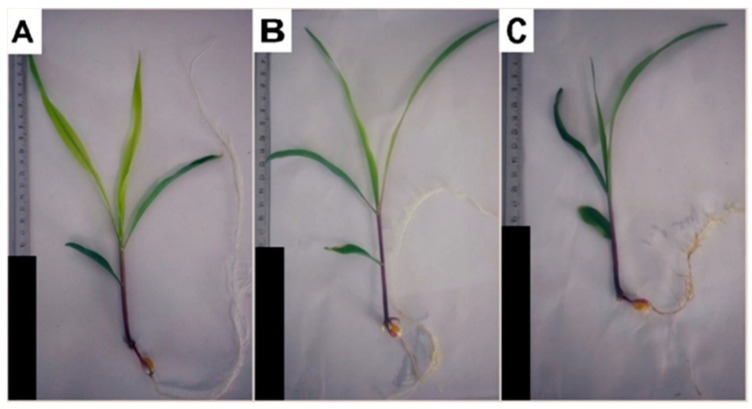
Hydroponically grown maize plants treated with 3-cyanobenzoic acid for 14 days: 0 mM (**A**), 0.5 mM (**B**), and 1.0 mM (**C**). Scale bars represent 10 cm.

Treatment with 3-cyanobenzoic acid significantly inhibited maize growth (Table 1). At 1.0 mM, plant height decreased by 20.3%. The leaf area was reduced by 21.4% at 0.5 mM and by 42% at 1.0 mM, while the culm diameter decreased by 26.5% at 0.5 mM and 34.9% at 1.0 mM. Shoot fresh biomass saw a 45.9% decrease at 1.0 mM compared to the control. Root length also showed significant reductions, with a 45.5% decrease at 0.5 mM and a striking 65.7% decrease at 1.0 mM. Moreover, fresh root biomass decreased by 62.9% at 1.0 mM. These decreases are illustrated in Figure 1. These substantial decreases underscore the significant impact of 3-cyanobenzoic acid on various growth parameters of maize.

### 3.2. Effects on SPAD Index, Chlorophyll a Fluorescence and Gas Exchange

**Figure 2:**
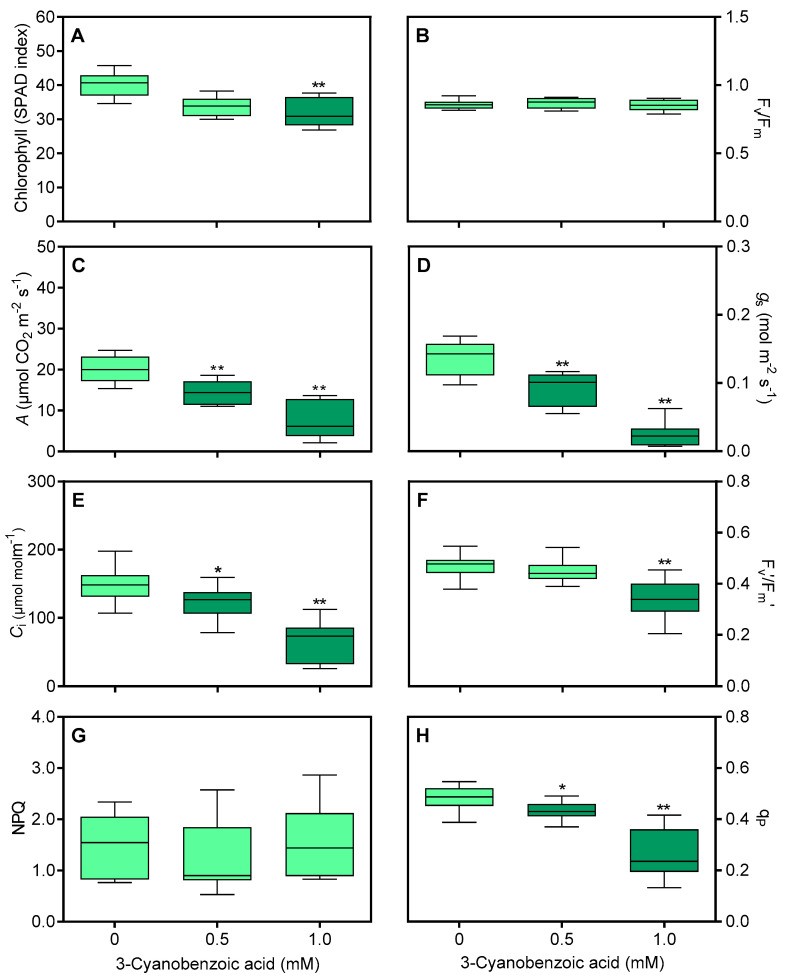
Effects of 3-cyanobenzoic acid on hydroponically grown maize plants for 14 days. Parameters measured include (**A**) chlorophyll content (SPAD index), (**B**) maximum quantum efficiency of PSII photochemistry (F_v_/F_m_), (**C**) net assimilation (*A*), (**D**) stomatal conductance (*g*_s_), (**E**) intercellular CO_2_ concentration (*C*_i_), (**F**) maximum efficiency of PSII (F_v′_/F_m′_), (**G**) non-photochemical quenching (NPQ), and (**H**) photochemical quenching (q_P_). Means values (n = 16–22 ± SEM) significantly different from the control are marked with * *p* ≤ 0.05, ** *p* ≤ 0.01, according to Dunnett’s test.

Maize plants grown with 3-cyanobenzoic acid for 14 d exhibited mild chlorosis. The total chlorophyll content, as indicated by the SPAD index in the second fully expanded leaf, decreased by 19.3% at 1.0 mM (Figure 2A). F_v_/F_m_ values remained unchanged (Figure 2B).

Under full photosynthetic conditions (1400 μmol m^−2^ s^−1^ irradiance and 400 µmol mol^−1^ CO_2_), several key parameters were significantly reduced compared to the controls. The *A* (Figure 2C) decreased by 23% to 0.5 mM and 56.7% to 1.0 mM. Similarly, *g*_s_ showed a marked reduction, decreasing by 32.8% at 0.5 mM and 68.2% at 1.0 mM (Figure 2D). The *C*_i_ also decreased by 21.4% at 0.5 mM and 44% at 1.0 mM (Figure 2E). In addition, the F_v′_/F_m′_ decreased by 24.8% at 1.0 mM (Figure 2F). While NPQ (Figure 2G) remained stable, q_P_ (Figure 2H) decreased by 10.9% at 0.5 mM and 39.3% at 1.0 mM.

**Figure 3:**
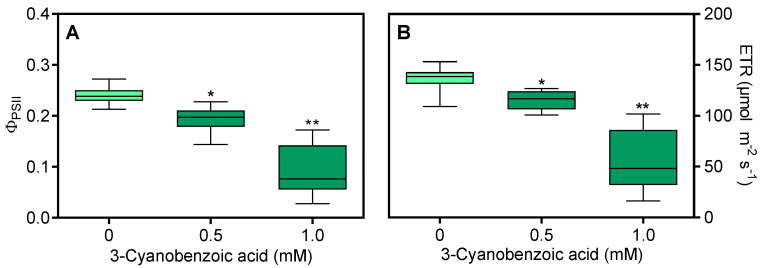
Effects of 3-cyanobenzoic acid on hydroponically grown maize plants for 14 days on quantum yield of photosystem II photochemistry (ϕ_PSII_) (**A**) and electron transport rate through PSII (ETR) (**B**). Means (n = 22 ± SEM) marked with * or ** are statistically different from the control according to Dunnett’s test at 5% and 1% significance levels, respectively.

The ϕ_PSII_ (Figure 3A) and ETR (Figure 3B) decreased by 15.3% (at 0.5 mM) and 51.4% (at 1.0 mM).

### 3.3. Effects on A/PAR Curve and Derived Parameters

**Figure 4:**
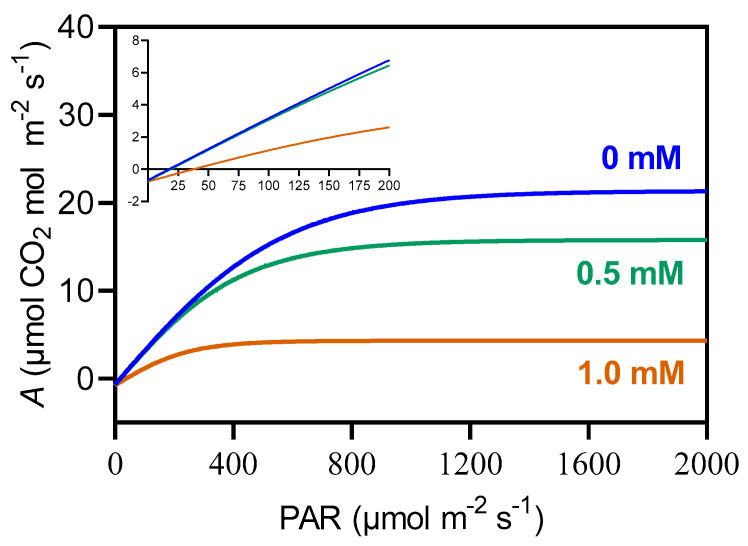
Average net assimilation (*A*) curves in response to varying photosynthetically active radiation (PAR) for maize plants grown hydroponically with 3-cyanobenzoic acid for 14 days. The initial linear region of the graph is magnified for clarity. Data are presented as mean values (n = 4).

Significant changes were seen in the *A* versus PAR (Figure 4). At 0.5 mM, *A* levels were already lower than those of the control. At 1.0 mM, the decline in *A* was more pronounced at higher PAR levels and in the slope of the first linear phase.

**Figure 5:**
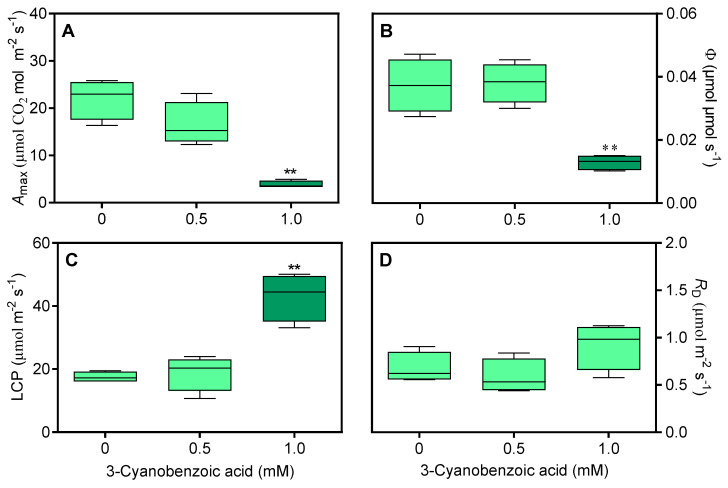
Effects of 3-cyanobenzoic acid on hydroponically grown maize plants after 14 days, focusing on parameters derived from the *A*/PAR curve: (**A**) net assimilation (*A*_max_), (**B**) apparent quantum yield (ϕ), (**C**) light compensation point (LCP), and (**D**) dark respiration rate (*R*_D)_. Means values (n = 3–4 ± SEM) significantly different from the control are marked with, ** *p* ≤ 0.01, according to Dunnett’s test.

The *A*_max_ decreased by a striking 82% at 1.0 mM (Figure 5A). The ϕ decreased by 57% at 1.0 mM (Figure 5B). Additionally, the LCP increased by 175% at 1.0 mM (Figure 5C), while *R*_D_ remained unaffected (Figure 5D).

### 3.4. Effects on A/C_i_ Curve and Derived Parameters

**Figure 6:**
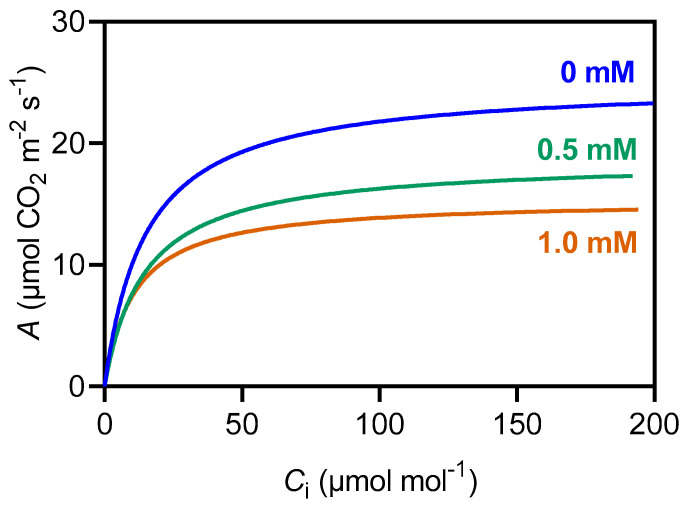
Average net assimilation (*A*) curves in response to varying intercellular CO_2_ concentration (*C*_i_) forma maize plants grown hydroponically with 3-cyanobenzoic acid for 14 days. Data are presented as mean values (n = 4–6).

To differentiate the effects of CO_2_ diffusion through the stomata, the *A* versus *C*_i_ curve was analyzed to derive key parameters, including V_cmax_, V_pmax_, *J*, and *g*_m_. The impact of 3-cyanobenzoic acid was clear at even low CO_2_ concentrations and became more pronounced at higher concentrations (Figure 6).

**Figure 7:**
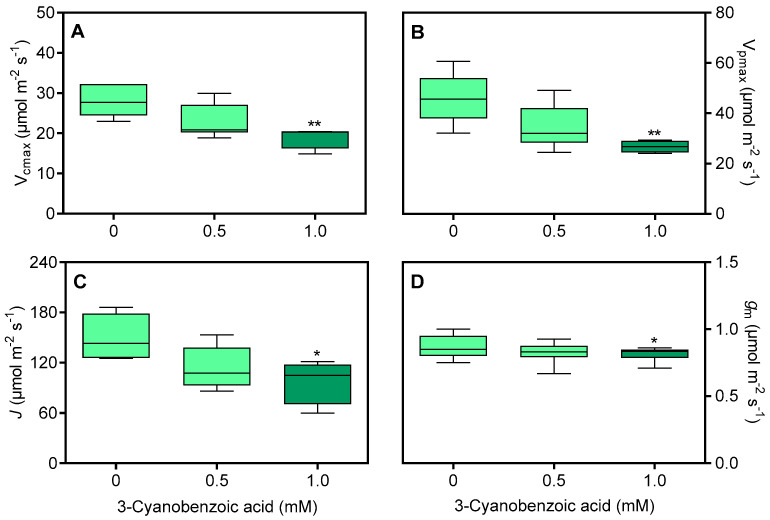
Effects of 3-cyanobenzoic acid on maize plants grown hydroponically for 14 days, focusing on parameters derived from the *A*/*C*_i_ curve: (**A**) maximum carboxylation rate of RuBisCo (V_cmax_), (**B**) maximum carboxylation rate of PEPCase (V_pmax_), (**C**) rate of photosynthetic electron transport (*J*), and (**D**) mesophyll conductance (*g*_m_). Means values (n = 4–6 ± SEM) significantly different from the control are marked with * *p* ≤ 0.05, ** *p* ≤ 0.01, according to Dunnett’s test.

At 1.0 mM, V_cmax_ decreased by 32% (Figure 7A), V_pmax_ by 42% (Figure 7B), *J* by 34.8% (Figure 7C), and *g*_m_ by 28% (Figure 7D).

### 3.5. Effects on Chlorophyll a Transient Curve and Derived Parameters

**Figure 8:**
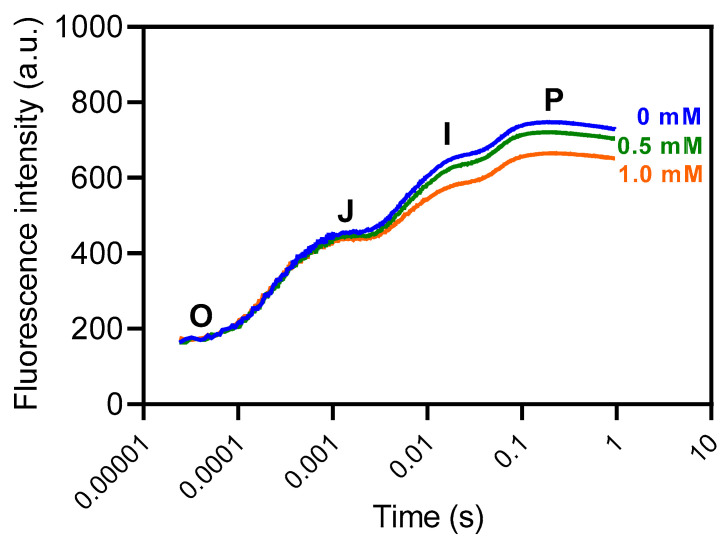
Chlorophyll *a* fluorescence OJIP transient curves in maize plants grown hydroponically with 3-cyanobenzoic acid for 14 days. The OJIP curve represents key fluorescence intensities: the minimal fluorescence when all PSII reaction centers are open (O step), the intensity at 0.002 s (J step), the intensity at 0.03 s (I step), and the maximal fluorescence when all PSII reaction centers are closed (P step, at 0.3 s). Data are presented as means (n = 18 ± SEM).

No significant changes were seen in the OJIP curve (Figure 8).

**Figure 9:**
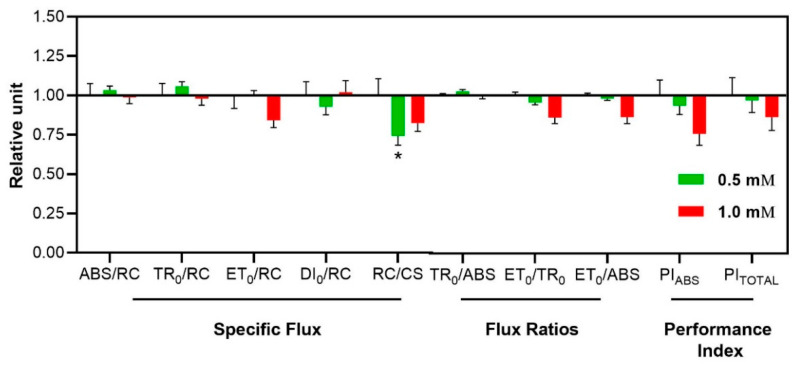
Effects of 3-cyanobenzoic acid on specific energy flux parameters in hydroponically grown maize plants after 14 days of treatment. Parameters include: absorption flux per reaction center (ABS/RC), energy trapping per reaction center (TR_0_/RC), electron transport per reaction center (ET_0_/RC), energy dissipation per reaction center (DI_0_/RC), reaction center density per cross-sectional area (RC/CS), quantum yield of primary PSII photochemistry (TR_0_/ABS), efficiency with which a trapped electron is transferred from Q_A_ to Q_B_ (ET_0_/TR_0_), quantum yield of electron transport from Q_A_ to Q_B_ (ET_0_/ABS), and performance indices (PI_ABS_ and PI_Total_). Data are presented as means (n = 18 ± SEM). Mean value significantly different from the control is marked with * *p* ≤ 0.05, according to Dunnett’s test.

Finally, a noteworthy reduction was seen in RC/CS, which decreased by 25.8% at 0.5 mM (Figure 9).

## 4. Discussion

Treatment with 3-cyanobenzoic acid resulted in a significant reduction in both foliar and root growth, as well as in key photosynthetic, photochemical, and carboxylative parameters in maize plants grown hydroponically for 14 days. These findings suggest that the compound impairs multiple aspects of plant development and function. Further steps aim to understand the reasons behind these declines and identify primary targets.

Our earlier study demonstrated significant damage to root growth and development in maize seedlings exposed to 3-cyanobenzoic acid for four days. This included reduced root length and biomass, a decrease in secondary roots, increased root respiration, heightened antioxidant activity, and elevated ROS production. While the mitochondrial respiratory chain was not directly affected, the observed damage was linked to membrane destabilization, deficiencies in nutrient and water absorption, and hormonal imbalances [23]. These effects are consistent with those observed in other plants exposed to monophenols, such as cinnamic and benzoic acids. Upon absorption, these acids undergo deprotonation, leading to the dissipation of the proton gradient and membrane depolarization. This cascade of events triggers an increase in ROS production and antioxidant activity, disrupting ion transport, nutrient absorption, and water balance [24,36,37,38]. Additionally, these disturbances extend to hormonal imbalances, stomatal closure, reduced chlorophyll content, diminished RuBisCo activity, and damage to photosynthetic reaction centers and electron transport chains [24].

Maize plants grown for 14 days exhibited damage like that observed in seedlings [23], suggesting an acclimatization response to unfavorable conditions. However, it is essential to determine whether the observed effects are solely due to continuous root damage or if there are also direct impacts on the aerial parts of the plant, including photosynthesis. Regardless of light intensity or CO_2_ supply, a clear reduction in net photosynthesis was observed. The negative correlation between compound concentration and photosynthetic performance—reflecting a trade-off between defense mechanisms and growth—is expected [39,40]. Consequently, the decrease in photosynthesis cannot be attributed solely to energy partitioning. A more comprehensive understanding of the underlying mechanisms requires the identification and integration of additional relevant parameters.

Various simple phenolics have been shown to impact photosynthetic parameters, with stomatal closure being one of the most common effects [37,41,42,43]. Typically, an increase in *C*_i_ is accompanied by a reduction in *g*_s_, signaling a decrease in the efficiency of carboxylation cycles. This has been demonstrated in soybean plants exposed to L-DOPA [44] and in poplar seedlings exposed to a phenolic mixture [43]. However, alternative patterns can also emerge. In cases of photosynthesis limitation due to stomatal closure, a decrease in *g*_s_ is often paired with a reduction in *C*_i_ [24]. Similar findings were observed in cucumber treated with cinnamic and benzoic acid derivatives [41]. As evidenced in the present study, this pattern suggests that the damage to diffusion processes is more severe than the impairment of the carboxylative systems (such as the C4 cycle, Hatch–Slack pathway, and C3 Calvin–Benson cycle). This observation, per se, provides a strong rationale for the noted decrease in *C*_i_, emphasizing the greater impact on stomatal function and CO_2_ diffusion.

Stomatal closure likely explains much of the observed reductions in photosynthetic parameters. By limiting CO_2_ availability within the leaf, this closure, combined with a decrease in *g*_m_, results in a substrate shortage for key enzymes such as PEPCase (V_pmax_) and RuBisCo (V_cmax_). This shortage compromises their carboxylative activities, which accounts for the observed declines in photosynthetic efficiency. Alternatively, 3-cyanobenzoic acid may have exerted a direct inhibitory effect on these enzymes. For instance, Gao et al. [38] showed that salicylic acid reduced RuBisCo activity and disrupted photochemical structures in *Chlamydomonas reinhardtii*. Similarly, Huang and Bie [45] reported that cinnamic acid decreased RuBisCo activity in *Vigna unguiculata* without affecting *C*_i_ or ϕ_PSII_, suggesting that direct enzyme inhibition could be a contributing factor in the present study.

Maize, a C4 plant, benefits from an efficient CO_2_ concentration mechanism that provides a distinct advantage over C3 plants. This mechanism is driven by PEPCase, which has a higher carboxylation rate than RuBisCo, enabling more effective CO_2_ fixation [1,2,3]. However, the observed decline in V_pmax_ was more pronounced than the reduction in V_cmax_, suggesting a compromised CO_2_ concentration mechanism. This impairment is further worsened by the lack of ribulose-1,5-bisphosphate (RuBP), the substrate for RuBisCo, which weakens the carboxylation process. The decreased plateau in the *A*/*C*_i_ curve shows a limitation in RuBP regeneration, a critical process for maintaining efficient photosynthesis [2,46,47]. RuBP regeneration relies heavily on ATP and NADPH, which are generated through the electron transport chain, as evidenced by the reduced ETR and the decrease in *J*. These findings underscore the impact of 3-cyanobenzoic acid on maize’s photosynthetic machinery, particularly on its ability to regenerate RuBP and sustain efficient carbon fixation. Although our results indicate significant disruptions in photosynthetic parameters, it is important to acknowledge that concluding 3-cyanobenzoic acid directly targets key photosynthetic pathways at this stage may be premature. The observed effects on photosynthesis, such as reductions in ETR, V_cmax_, V_pmax_, and chlorophyll content, could also be indirect consequences of oxidative stress, which is known to impair photosynthetic machinery through ROS accumulation and membrane destabilization. To confirm whether the primary target of 3-cyanobenzoic acid is within photosynthetic pathways, further studies are required. A combination of omics approaches, such as transcriptomics and proteomics, would allow us to identify changes in the expression and abundance of proteins involved in photosynthesis and oxidative stress responses [48]. For example, decreased levels of PPDK, RuBisCo, or other Calvin cycle enzymes could point to direct inhibition. Additionally, metabolomics could reveal disruptions in ATP and NADPH production, as well as in the pools of photosynthetic intermediates. Complementary in silico molecular docking studies could help predict the binding affinity of 3-cyanobenzoic acid to target enzymes, such as RuBisCo, PPDK, or components of the electron transport chain. Such simulations would provide insights into the likelihood of direct enzymatic inhibition. Moreover, targeted enzymatic assays would be essential to confirm the direct inhibition of photosynthetic enzymes, distinguishing them from generalized oxidative stress effects. These combined approaches would clarify the mode of action of 3-cyanobenzoic acid and determine whether it is a direct or indirect disruptor of photosynthesis.

Photosynthesis involves the capture of light energy and its conversion into chemical energy, processes that are reflected in key parameters such as q_P_, F_v′_/F_m′_, ϕ_PSII_, and ETR. The observed changes in these parameters show a significant reduction in ATP and NADPH production, suggesting an energy deficit that hampers the plant’s ability to conduct essential metabolic functions. Additionally, the insufficient dissipation of energy impacts the NPQ mechanism, leading to lower ROS production in leaves compared to roots [23]. Several phenolic compounds, including *O*-methoxybenzoic, benzoic, usnic, salicylic, cinnamic, syringic, and phthalic acids, are known to interfere with antenna complexes, disrupting the function of reaction centers and blocking electron transport between Q_A_ and Q_B_. These disruptions reduce photochemical efficiency and increase heat dissipation, as shown in earlier studies [24,38,42,43]. Similarly, photochemical quenching parameters change similarly to *A* in plants under growth stress, where ATP and NADPH consumption are critical. Variations in carboxylation efficiency, CO_2_ availability, or carbohydrate export from cells can further influence this consumption, highlighting the complex interdependencies within the photosynthetic process [49].

To evaluate potential structural damage to the photochemical process, chlorophyll *a* fluorescence is measured in the dark, when the carboxylation cycle is inactive. Key parameters such as F_v_/F_m_, SPAD index, OJIP curve, and JIP test are critical for this assessment. F_v_/F_m_ reflects the maximum efficiency of photosystem II, indicating the proportion of open reaction centers. The lack of change in F_v_/F_m_ suggests that there was no severe damage to photochemical structures, as the parameter remains stable even with reduced chlorophyll content [29,38]. However, while the unaltered F_v_/F_m_ indicates that 3-cyanobenzoic acid did not severely damage the photochemical machinery, the observed decrease in reaction centers per cross-section (RC/CS) and total chlorophyll levels suggests that some degree of damage may have occurred, potentially affecting overall photosynthetic efficiency.

In their study on the effects of various phenolic compounds on the photosynthesis of *C. reinhardtii*, Gao et al. [38] found that while benzoic acid did not affect F_v_/F_m_, it significantly altered chlorophyll and carotenoid levels, as well as the OJIP curve and JIP-test parameters. Specifically, benzoic acid reduced the RC/CS ratio, like 3-cyanobenzoic acid, and affected PI_ABS_, an important indicator of PSII photoinhibition under abiotic stress, while also enhancing the J phase in the OJIP curve. These changes closely resemble those caused by DCMU, a PSII inhibitor that disrupts electron transport between plastoquinones, thereby reducing PSII quantum efficiency by disconnecting smaller antenna molecules and blocking electron flow beyond Q_A_. Based on these observations, it is likely that 3-cyanobenzoic acid induces photochemical damage to PSII reaction centers by interfering with antenna molecules, leading to a reduction in chlorophyll content. This decrease in chlorophyll is a primary effect of phenolic compounds on photosynthesis, likely due to either decreased biosynthesis or increased degradation of chlorophyll [24]. Additionally, carotenoids, which function as accessory pigments in antenna complexes, may also be reduced because of hormonal imbalances, potentially linked to an increase in abscisic acid, a carotenoid-derived hormone [50].

The effects of 3-cyanobenzoic acid on maize seedling roots suggest a disruption in hormonal balance [23]. Abscisic acid (ABA) is known to induce stomatal closure [40,51], but certain phenolic compounds, such as hydroxybenzoic and hydroxycinnamic acids, can counteract this effect. Similarly, 3-cyanobenzoic acid, like 3-hydroxybenzoic acid, exhibits diverse and complex impacts on plant physiology [24,52]. Monosubstituted benzoic acids, like 3-cyanobenzoic acid, can inhibit ethylene synthesis at high concentrations, while some monophenols stimulate auxin degradation [24]. Given these effects, it is plausible to hypothesize that 3-cyanobenzoic acid may contribute to root damage and hinder maize growth and photosynthesis. This could be linked to elevated ABA levels relative to other hormones, which may lead to reduced stomatal conductance and altered carotenoid synthesis. Such changes in hormonal regulation could help explain the observed damage to reaction centers, further impairing the plant’s ability to efficiently capture light and perform photosynthesis.

Lastly, the LCP, which is the minimum irradiance needed to sustain basal metabolism, showed the most significant change [34]. The increase in LCP can be attributed to a combination of reduced carbon assimilation, impaired quantum efficiency, and elevated root respiration [23]. Despite the challenges imposed by 3-cyanobenzoic acid, maize plants exhibited a degree of adaptability, adjusting their metabolic processes to cope with the altered growth conditions. However, in environments with insufficient light, maize plants remain vulnerable, as they are unable to meet the high demands of basal metabolism. At high irradiance, NPQ would likely be inadequate to prevent the formation of ROS in the aerial parts of the maize plants, further exacerbating the stress.

Future research on 3-cyanobenzoic acid should prioritize elucidating its biochemical targets, particularly PPDK, through comprehensive enzymatic assays, and determining its selectivity between C4 weeds and C3 crops. Field trials are essential to assess its efficacy, stability, and interactions within the environment, including its soil adsorption and bioavailability. Exploring chemical modifications could improve both its potency and safety profile, while in-depth toxicity evaluations and advanced formulation strategies will be critical for optimizing its use as a preemergence herbicide. Despite its promising potential, several challenges remain, including environmental persistence, high development costs, stringent regulatory hurdles, and the risk of resistance development. A strategic, multidisciplinary approach is vital to addressing these challenges effectively. These factors will play a crucial role in shaping future studies and advancing the compound’s development toward practical application.

## 5. Conclusions

The presence of 3-cyanobenzoic acid significantly hampers maize growth by disrupting photosynthetic, photochemical, and carboxylative processes. The primary driver of reduced photosynthesis is root damage, which diminishes both stomatal and mesophyll conductance, thereby limiting CO_2_ availability for RuBisCo and PEPCase activity. This disruption in CO_2_ assimilation is compounded by damage to the antenna complexes and reaction centers of photosystem II, likely caused by reduced chlorophyll content and a possible diversion of carotenoids for ABA synthesis, which triggers stomatal closure. While maize plants exhibit some resilience to 3-cyanobenzoic acid, their vulnerability becomes evident under low-light conditions due to the high metabolic costs associated with maintaining basic physiological functions. Under high light conditions, the inadequacy of the photosynthetic machinery leads to increased oxidative stress, further exacerbating growth limitations. These findings highlight the potential of 3-cyanobenzoic acid and its derivatives as herbicide candidates, targeting key photosynthetic pathways. By leveraging advanced screening technologies and analyzing plant-specific responses, such as those observed in maize, we can develop selective and environmentally sustainable herbicides. Evaluating the phytotoxicity of potential plant protection products is essential to ensuring their effectiveness against target weeds while minimizing adverse effects on non-target species and ecosystems. These assessments provide valuable insights into the compounds’ mechanisms of action, selectivity, and safety, facilitating the development of effective and sustainable agricultural solutions.

## Figures and Tables

**Table 1:** Effects of 3-cyanobenzoic acid on maize plant growth during a 14-day hydroponic cultivation period.

	3-Cyanobenzoic Acid (mM)		
	0	0.5	1.0
Plant height (cm)	9.51 ± 0.27	8.77 ± 0.26	7.57 ± 0.20 **
Shoot fresh weight (g)	1.11 ± 0.12	0.88 ± 0.06	0.60 ± 0.05 **
Leaf area (cm^2^)	38.0 ± 2.84	29.9 ± 1.71 *	22.0 ± 1.38 **
Culm diameter (cm)	3.34 ± 0.19	2.46 ± 0.08 **	2.17 ± 0.08 **
Root length (cm)	28.7 ± 1.77	15.4 ± 0.81 **	9.84 ± 0.47 **
Root fresh weight (g)	0.33 ± 0.04	0.25 ± 0.02	0.12 ± 0.01 **

Means values (n = 18 ± SEM) significantly different from the control are marked with * *p* ≤ 0.05, ** *p* ≤ 0.01, according to Dunnett’s test.

## Data Availability

Data are contained within the article.

## Abbreviations

The following abbreviations are used in this manuscript:

*A*	net assimilation
ABS/RC	absorption flux per reaction center at t = 0 or a measure for an average antenna size
*A*_max_	net assimilation rate
*C*_i_	intercellular CO_2_ concentration
DI_0_/RC	energy dissipation per reaction center
*E*	transpiration rate
ETR	electron transport rate through PSII
ET_0_/ABS	quantum yield for electron transport (at t = 0) from Q_A_ to Q_B_
ET_0_/RC	electron transport flux per reaction center at t = 0
ET_0_/TR_0_	probality (at t = 0) of a trapped exciton transfer an electron beyond Q_A_^−^ into the ETR
F_0_	minimal fluorescence intensity when all the reaction centers of PSII are open
F_m_	maximum fluorescence intensity when all reaction centers of PSII are closed
F_v_	variable fluorescence
F_v_/F_m_	maximum quantum yield of PSII photochemistry
F_v′_/F_m′_	maximum quantum yield of PSII photochemistry in light
*g*_m_	mesophilic conductance
*g*_s_	stomatal conductance
*J*	photosynthetic electron transport rate
LCP	light compensation point
NPQ	non-photochemical quenching
OJIP	chlorophyll *a* fluorescence transient with O-J-I-P phases
PAR	photosynthetically active radiation
PEPCase	phosphoenolpyruvate carboxylase
PI_ABS_	indicator of the conservation of energy absorbed by PSII until the decrease of the Q_B_
PI_TOTAL_	indicator of energy conservation in PSII up to the reduction of final PSI acceptors.
PPDK	pyruvate *O*-phosphate dikinase
Q_A_	primary quinone acceptor of PSII
Q_B_	secondary quinone acceptor of PSII
q_P_	photochemical quenching coefficient
RC/CS	amount of active PSII reaction centers per cross-section
*R*_D_	dark respiration rate
RuBisCo	ribulose-1,5-bisphosphate carboxylase/oxygenase
TR_0_/ABS	maximum quantum yield of primary photochemistry at t = 0
TR_0_/ABS	quantum efficiency of primary PSII photochemistry
TR_0_/RC	trapped energy flux per reaction center at t = 0
V_cmax_	maximum RuBisCo carboxylation rate
V_pmax_	maximum PEPCase carboxylation rate
ϕ	apparent quantum yield
ϕ_PSII_	effective quantum yield of PSII photochemistry
